# Feasting and Mobility in Iron Age Ireland: Multi-isotope analysis reveals the vast catchment of Navan Fort, Ulster

**DOI:** 10.1038/s41598-019-55671-0

**Published:** 2019-12-24

**Authors:** Richard Madgwick, Vaughan Grimes, Angela L. Lamb, Alexandra J. Nederbragt, Jane A. Evans, Finbar McCormick

**Affiliations:** 1 Cardiff University, Cardiff, UK; 2 Memorial University Newfoundland, St. John’s, Canada; 3 National Environmental Isotope Facility, British Geological Survey, Nottingham, UK; 4 Queen’s University Belfast, Belfast, UK

**Keywords:** Biogeochemistry, Biogeochemistry

## Abstract

Navan Fort is an iconic prehistoric Irish ceremonial centre and the legendary capital of Ulster. The fort has produced an exceptional pig-dominated faunal assemblage that also contained a barbary macaque skull. Dating from the 4^th^ to 1^st^ century BC, it is likely to be a ceremonial feasting centre that may have drawn people and their animals from across Ulster and beyond. This study uses a multi-isotope (^87^Sr/^86^Sr, δ^34^S, δ^13^C, δ^15^N) approach to identify non-local animals and reconstruct site catchment. New biosphere mapping means that isotope data can be more confidently interpreted and the combination of strontium and sulphur analysis has the potential to estimate origins. In the absence of human remains, fauna provide the best proxy for human movement. Results for the 35 analysed animals are wide-ranging, especially in terms of strontium (0.707–0.715), which has the largest range for an Irish site. Sulphur values are more restricted (13.1‰−17.1‰) but are high in the context of British and Irish data. Results provide clear evidence for animals (and thus people) coming from across Ulster and beyond, demonstrating the site’s wide catchment. Navan Fort was clearly a major ceremonial centre with far-reaching influence and hosted feasts that drew people and animals from afar.

## Introduction

**Figure 1:**
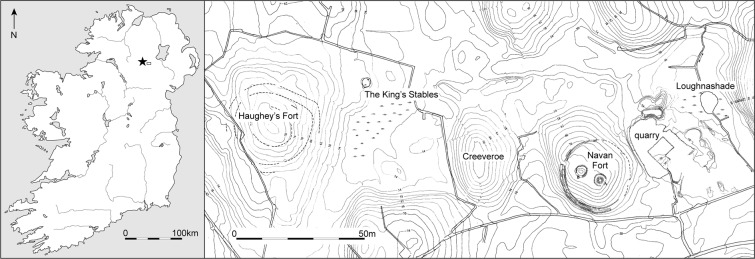
Map showing the location of Navan Fort. Modified using Adobe Illustrator CC with permission from Queen’s University Belfast, CAF Data Structure Report 13.

Navan Fort is one of the great later prehistoric regional ceremonial centres of Ireland which are documented in later historical and mythological sources^1,2^. While largely abandoned by the early medieval period these sites, which include Tara, Rath Croghan and Dūn Ailinne, continued to have strong associations with kingship and power throughout the medieval period. Navan Fort, the Emain Macha of the early texts, was considered to be the ancient capital of Ulster, the northern part of Ireland^1,2^ (Fig. 1). The site consists of a large circular enclosure, 250 m in diameter, with the main interior feature comprising a large mound (Site B). This covered a large circular ceremonial wooden structure, 40 m in diameter, which Warner^3^ refers to as a temple. Both date to the 1^st^ century BC^4^. Artefactual evidence, however, indicated settlement on the site dating as early as the Neolithic^5^ and excavation revealed a series of round buildings dating to the end of the Bronze Age and Early Iron Age. The 40 m structure at Navan and the amphitheatre-like structures at Dūn Ailinne^6^ imply ceremonial buildings built to accommodate large numbers of people. Based on their size, the implication is that the centres catered for regional or provincial, rather than just local populations. They may have provided lynchpins in the landscape and centres for wide-ranging connectivity, both from within Ulster and perhaps beyond.

Only a single human clavicle was recovered from the site and therefore animal remains provide the best proxy for human movement and provide a key resource for reconstructing the site’s catchment. A substantial pig-dominated faunal assemblage (NISP: 2624) representing a minimum of 104 animals, was recovered from the site^7^. The most startling find was the skull of a barbary macaque^8^ that must have come from at least as far as southern Iberia, providing further evidence for the high-status nature of the site and suggesting long distance contact (albeit not necessarily direct). The high proportion of pig remains (63% of NISP compared with 30% cattle and 8% sheep/goat^7^) is very rare for Iron Age Britain and Ireland^9,10^. This suggests that Navan Fort is likely to have been a feasting centre, as pigs are well-suited as feasting animals, and in early Irish literature pork is the preferred food of the feast^11^. This is the case as they gain weight rapidly, have large litters and can be fed a broad range of produce, meaning raising meat for the feast can be achieved rapidly and efficiently. In addition, they do not produce milk or wool and therefore large stocks can be slaughtered without harming a secondary product economy. Pigs are well-suited to household husbandry due to their omnivorous diet, meaning they can be raised on meal scraps and even human faeces. They may therefore provide a good proxy for human movement, as they are more likely to be brought as a contribution to the feast by those that raised them^9^, whereas cattle and sheep are more likely to be raised by specialist producers. There is, in any case, no strong evidence for specialist production in Early Iron Age Ireland and therefore it is highly likely that these animals were brought on the hoof, by members of the household that raised them. The isotope analysis of pig, cattle and sheep bones from Navan Fort has the potential to test this hypothesis and to reconstruct the catchment of the site.

Strontium (^87^Sr/^86^Sr) and sulphur (δ^34^S) isotope ratios are analysed to identify non-local animals and explore patterns of movement. Strontium isotope analysis has become a common method for investigating childhood origins in humans and animals. ^87^Sr/^86^Sr isotope ratios essentially reflect the underlying lithology of the area from where an individual’s food derives, although superficial deposits such as tills, loess and peat also affect local signals^12^. The diverse geology of Britain and Ireland means this approach is well suited to exploring mobility. Bone and dentine are diagenetically affected and therefore dental enamel is analysed, providing a more temporally resolved indication of origins for the period when the sampled tooth was mineralising. Variation in sulphur isotope values has a more diverse aetiology and is affected by lithology, atmospheric deposition and hydrological conditions^13^. It has not been as widely used in provenancing studies and biosphere mapping is not as advanced as for strontium, but it has been shown to be a useful discriminant as part of a multi-isotope methodology^14^. In an Irish context coastal proximity has been shown to be an important driver^15^. Sulphur isotope analysis is undertaken on collagen from bone and dentine. In the case of bone, from which most samples were taken, this provides a time-averaged signal for the animal’s life in comparison to the shorter time period represented by the analysis of enamel. However, in short-lived animals such as these (especially pigs), the temporal range is certain to overlap and values should be comparable. These data are augmented by carbon (δ^13^C) and nitrogen (δ^15^N) isotope analyses to assess marine dietary influence on provenancing proxies and to investigate husbandry regimes. These approaches have been introduced extensively elsewhere^16^ and are long established methods for reconstructing human and animal diets. In addition, strontium and sulphur isotope analysis of seven modern plants from the vicinity of Navan Fort provide data on the locally bioavailable range. Details of the samples are presented in Table 1.

**Table 1:** Details of the samples from Navan Fort.

Sample	Context	Taxon	Bone/dentine sample	Side	Tooth sample	Tooth surface	Lobe	Location
NAV01	152	Pig	Mandible	L	M2	Lingual	Mesial	REJ-mid cusp
NAV02	158	Pig	Mandible	L	M2	Lingual	Distal	REJ-mid cusp
NAV03	108	Pig	Mandible	L	M2	Lingual	Mesial/Distal	REJ-mid cusp
NAV04	B3	Pig	Mandible	L	M2	Lingual	Mesial	REJ-mid cusp
NAV05	152	Pig	Mandible	R	M2	Lingual	Mesial	REJ-mid cusp
NAV06	152	Pig	Mandible	R	M2	Lingual	Mesial	REJ-mid cusp
NAV07	13	Pig	Mandible	R	M2	Lingual	Distal	REJ-mid cusp
NAV08	242	Pig	Mandible	L	M2	Lingual	Mesial	REJ-mid cusp
NAV09	150	Pig	Mandible	L	M2	Lingual	Mesial	REJ-mid cusp
NAV10	5	Pig	Mandible	R	M2	Lingual	Mesial	REJ-mid cusp
NAV11	303	Pig	Mandible	L	M2	Lingual	Mesial	REJ-mid cusp
NAV12	152	Pig	Mandible	L	M2	Lingual	Mesial	REJ-mid cusp
NAV13	183	Pig	Mandible	R	M2	Buccal	Mesial	REJ-mid cusp
NAV14	598	Pig	Mandible	R	M2	Lingual	Distal	REJ-mid cusp
NAV15	152	Pig	Mandible	R	M2	Buccal	Distal	REJ-mid cusp
NAV16	133	Pig	Mandible	L	M2	Lingual	Distal	REJ-mid cusp
NAV17	598	Pig	Mandible	R	M2	Lingual	Mesial	REJ-mid cusp
NAV18	502	Cattle	Mandible	L	M2	Buccal	Distal	Upper cusp
NAV19	580	Cattle	Mandible	L	M1	Lingual	Distal	Lower cusp
NAV20	605	Cattle	Mandible	L	M1	Buccal	Mesial	Lower cusp
NAV21	502	Cattle	Mandible	R	M2	Buccal	Distal	Upper cusp
NAV22	5	Cattle	Maxilla	R	M2	Lingual	Distal	Mid cusp
NAV23	152	Cattle	Maxilla	R	M2	Buccal	Distal	Lower cusp
NAV24	280	Cattle	Mandible	L	M1/2	Distal	Distal	Mid cusp
NAV25	598	Cattle	Maxilla	L	M3	Buccal	Distal	Upper cusp
NAV26	151	Cattle	Upper premolar	L	dp4	Lingual	Mesial	Lower cusp
NAV27	J	Caprine	Mandible	R	M1	Lingual	Mesial	Mid cusp
NAV28	3	Caprine	Mandible	L	M1	Lingual	Mesial	Mid cusp
NAV29	—	Pig	Maxilla	R	M2	Buccal	Distal	Mid cusp
NAV30	—	Pig	Maxilla	R	M2	Lingual	Distal	Mid cusp
NAV35	151	Pig	—	L	M2	Buccal	Distal	Mid cusp
NAV36	158	Pig	—	L	M2	Buccal	Distal	Mid cusp
NAV37	152	Pig	—	R	M2	Buccal	Distal	Mid cusp
NAV38	152	Pig	—	L	M2	Buccal	Distal	Mid cusp
NAV39	A2	Pig	—	R	M2	Buccal	Distal	Mid cusp

## Results

**Figure 2:**
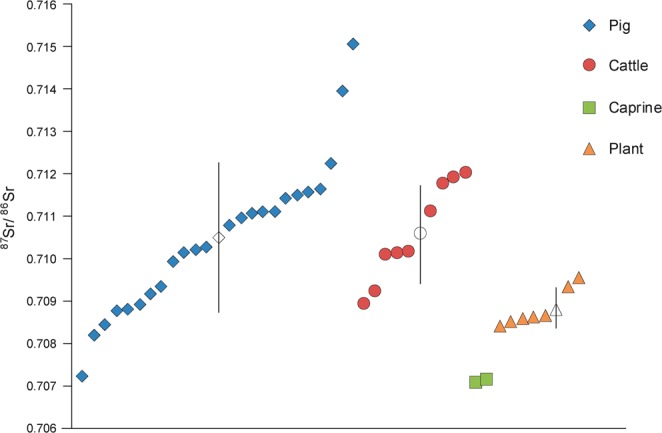
Strontium isotope results plotted in ascending order for each taxon (presented in addition to the bivariate plot in Fig. 3, as not all samples have sulphur isotope values). Unshaded markers represent the mean with error bars showing 1σ.

**Figure 3:**
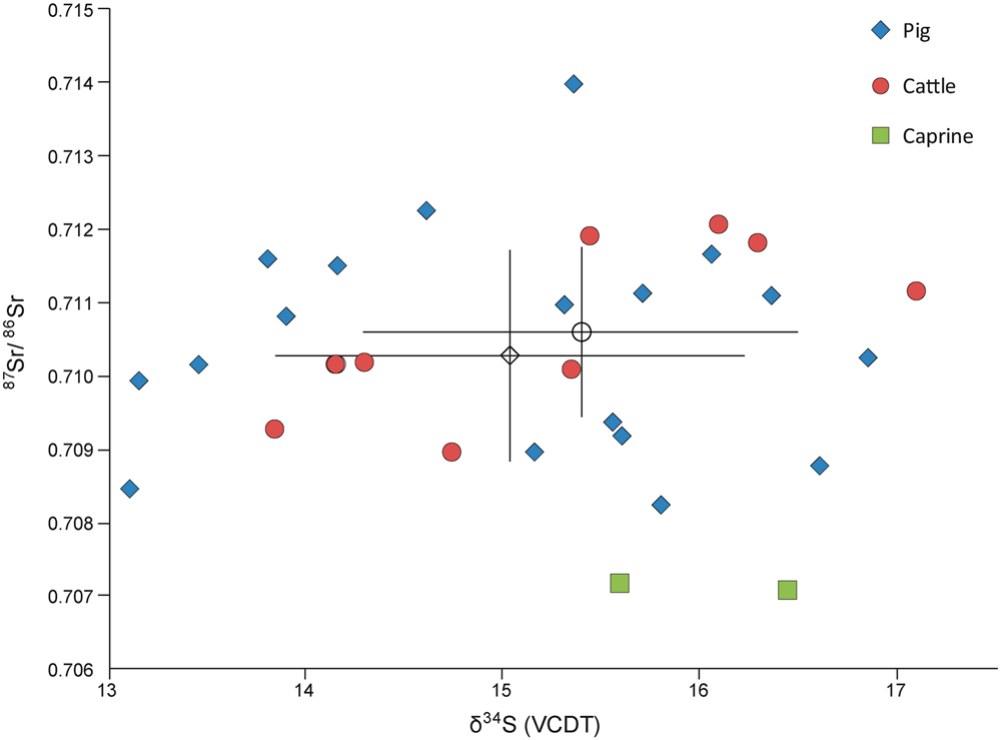
Strontium (^87^Sr/^86^Sr) isotope results plotted against sulphur (δ^34^S) isotope data. Unshaded markers represent the mean with error bars showing 1σ.

**Figure 4:**
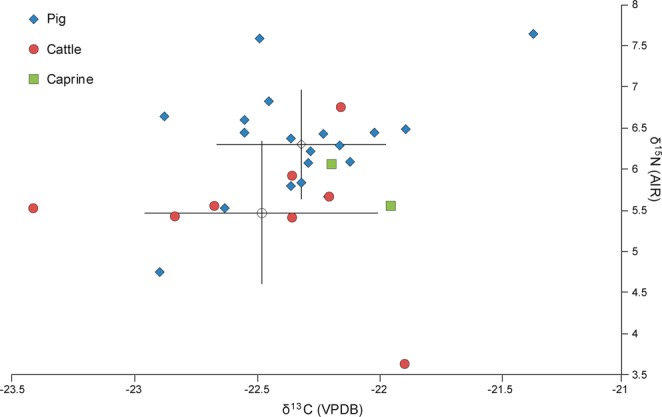
Carbon (δ^13^C) and nitrogen (δ^15^N) isotope results. Unshaded markers represent the mean with error bars showing 1σ.

Results are presented in Tables 2 and 3 and Figs. 2–4. Results are described by separate isotope systems (strontium, sulphur and carbon/nitrogen) and discussed in terms of provenance and husbandry. Plant isotope data are presented in the discussion to inform the interpretation of faunal data. Shapiro-Wilk tests demonstrated that all pig and cattle datasets were normally distributed and therefore means and standard deviations are presented on graphs.

**Table 2:** Isotope (^87^Sr/^86^Sr, δ^34^S, δ^13^C, δ^15^N) data with quality control indicators. NAV35–39 were only analysed for strontium isotope data. Other missing values relate to C:N and N:S ratios being outside acceptable ranges. % Coll refers to the collagen yield.

Sample	Taxon	δ^15^N	δ^13^C	δ^34^S	%N	%C	%S	C:N	C:S	N:S	% Coll	^87^Sr/^86^Sr
NAV01	Pig	5.8	−22.4	13.5	12.4	34.1	0.14	3.3	635	198	18.1	0.710152
NAV02	Pig	6.4	−22.5	14.2	12.3	34.7	0.14	3.3	660	201	8.2	0.711505
NAV03	Pig	6.6	−22.9	15.8	7.7	21.8	0.12	3.3	484	147	3.9	0.708233
NAV04	Pig	5.7	−22.2	13.9	13.3	36.8	0.2	3.2	490	152	15.0	0.710808
NAV05	Pig	6.6	−22.5	16.6	12.9	35.9	0.13	3.2	736	227	12.7	0.708791
NAV06	Pig	6.8	−22.4	13.2	10.6	30.5	0.16	3.4	508	151	5.5	0.709934
NAV07	Pig	5.5	−22.6	13.1	14.2	39.5	0.14	3.3	753	232	15.1	0.708471
NAV08	Pig	6.5	−21.9	16.9	12.4	34.6	0.17	3.3	543	166	10.6	0.710252
NAV09	Pig	6.4	−22	15.2	11	30.8	0.15	3.3	547	167	10.8	0.708956
NAV10	Pig	6.4	−22.4	15.7	13.5	38	0.17	3.3	595	182	17.3	0.71114
NAV11	Pig	7.6	−21.4	15.6	13.4	37.7	0.13	3.3	774	236	8.8	0.709194
NAV12	Pig	6.1	−22.3	16.4	11.3	31.9	0.15	3.3	567	173	11.1	0.711099
NAV13	Pig	7.6	−22.5	16.1	12.4	34.8	0.16	3.3	580	177	11.8	0.711669
NAV14	Pig	6.3	−22.2	15.4	12.5	34.9	0.17	3.3	547	169	10.9	0.713979
NAV15	Pig	6.1	−22.1	15.6	12.3	34.8	0.19	3.3	489	147	8.4	0.709365
NAV16	Pig	6.2	−22.3	15.3	12.2	34.4	0.15	3.3	612	187	10.6	0.710983
NAV17	Pig	5.8	−22.3	13.8	14.5	41.3	0.21	3.3	525	158	11.6	0.711603
NAV18	Cattle	5.4	−22.8	16.3	11.4	32.5	0.16	3.3	542	163	4.7	0.711785
NAV19	Cattle	5.5	−22.7	16.1	6.6	18.9	0.1	3.4	504	150	6.5	0.71203
NAV20	Cattle	3.6	−21.9	17.1	13	36.7	0.18	3.3	543	164	17.0	0.711135
NAV21	Cattle	5.5	−23.4	15.4	7.9	23	0.13	3.4	471	140	4.4	0.710078
NAV22	Cattle	5.9	−22.3	14.2	13.7	38.4	0.18	3.3	569	174	14.0	0.710144
NAV23	Cattle	5.7	−22.2	13.9	13.2	37.5	0.17	3.3	588	177	10.0	0.709263
NAV24	Cattle	—	—	—	—	—	—	—	—	—	—	0.71016
NAV25	Cattle	5.4	−22.3	14.8	13.9	38.9	0.18	3.3	576	176	17.1	0.708951
NAV26	Cattle	6.7	−22.1	15.5	11	31.3	0.15	3.3	556	168	ND	0.711903
NAV27	Caprine	6	−22.2	15.6	12.6	35.9	0.18	3.3	531	160	16.3	0.707142
NAV28	Caprine	5.5	−21.9	16.5	11.5	32.5	0.15	3.3	577	175	6.6	0.707067
NAV29	Pig	6.4	−22.2	14.6	14.5	40.8	0.18	3.3	605	184	9.5	0.712271
NAV30	Pig	4.8	−22.9	—	9.2	27.4	—	3.5	—	—	11.7	0.707252
NAV35	Pig	—	—	—	—	—	—	—	—	—	—	0.710152
NAV36	Pig	—	—	—	—	—	—	—	—	—	—	0.711505
NAV37	Pig	—	—	—	—	—	—	—	—	—	—	0.708233
NAV38	Pig	—	—	—	—	—	—	—	—	—	—	0.710808
NAV39	Pig	—	—	—	—	—	—	—	—	—	—	0.708791

**Table 3:** Summary statistics for the isotope results.

		Pigs	Cattle	Caprines
^87^Sr/^86^Sr	N	24	9	2
	Mean	0.710509	0.710594	0.707094
	1σ	0.001789	0.001154	—
	Median	0.710544	0.710150	—
	IQR	0.002085	0.001707	—
δ^34^S	N	18	8	2
	Mean	15.0	15.4	16.0
	1σ	1.2	1.1	—
	Median	15.3	15.4	—
	IQR	1.8	1.6	—
δ^13^C	N	19	8	2
	Mean	−22.3	−22.5	−22.1
	1σ	0.3	0.5	—
	Median	−22.3	−22.3	—
	IQR	0.3	0.5	—
δ^15^N	N	19	8	2
	Mean	6.3	5.5	5.8
	1σ	0.7	0.9	—
	Median	6.4	5.5	—
	IQR	0.6	0.3	—

The results from strontium isotope analysis are very wide-ranging. The large sample of pigs has by far the greatest range, with values from 0.7072 to 0.7150 (Table 3, Fig. 2). This is one of the largest ranges recorded for fauna from Britain and Ireland. It is also noteworthy that little clustering is apparent in the dataset. Only a single pig has a value <0.708 and only three have values >0.712 (0.7123, 0.7140 and 0.7150), meaning the vast majority of pigs (n = 20) range from 0.708 to 0.712. The nine cattle show a more limited range of 0.7089 to 0.7120. The graph shows that evidence for clustering is again weak, although samples were too small for K-means cluster analysis to be undertaken. It is noteworthy that both caprines have lower values (<0.7072) than all other animals in the dataset.

The sulphur isotope dataset is smaller as two samples had poor N:S ratios and the five that were previously analysed^14^ were not subject to sulphur analysis. The results have a much more limited range in relative terms, but are dominated by high values, with all exceeding 13‰. The pig range is again the largest (13.1‰ to 16.9‰) and comprises the four lowest values in the dataset (from 13.1‰ to 13.8‰). The spread of results is again well balanced, with the graph displaying weak evidence for a bimodal distribution (Fig. 3). Six animals have values <14.2‰ and 11 have values >15.1‰, with only a single sample sitting between these groups, but this certainly does not constitute a clear pattern of distribution, especially given the limited overall range. The cluster with higher sulphur values comprises diverse strontium values and has no clear positive or negative trend. However, the cluster with lower sulphur values shows a positive trend with strontium results that continues to the sample that sits between the clusters. This pattern is noteworthy, but relates to only seven samples. The cattle also show an even spread of values. There are fewer low values (one <14.2‰) and the dataset also comprises the highest value (17.1‰). The two caprine samples are towards the upper end of the range (15.6‰ and 16.5‰).

The carbon and nitrogen isotope data shows limited variation (Table 3, Fig. 4). As omnivores, pigs can have very diverse δ^15^N isotope values, but the range at Navan Fort is restricted, from 4.8‰ to 7.4‰. In spite of these relatively low values, the pig mean of 6.3‰ is still higher than for other taxa and the three highest values are all from pigs. The cattle sample also has a limited range, with all but one of the samples ranging from 5.4‰ to 6.7‰. A single outlier has a value of 3.6‰. The two caprine samples align closely with the bulk of the cattle dataset. Carbon isotope values also show little variation and are characteristic of a C3 ecosystem. The majority of animals cluster between −22.9‰ and −21.9‰. There is very little evidence for inter-taxonomic variation, but two outliers are noteworthy. A pig has the highest δ^13^C and joint highest δ^15^N value in the dataset (−21.4‰ and 7.6‰). By contrast a cattle sample has the lowest value of −23.4‰, but is typical in terms of its nitrogen value (5.5‰). There is no evidence for a linear relationship between carbon and nitrogen, carbon and sulphur or nitrogen and sulphur in the dataset.

## Discussion

### Defining local bioavailability

**Figure 5:**
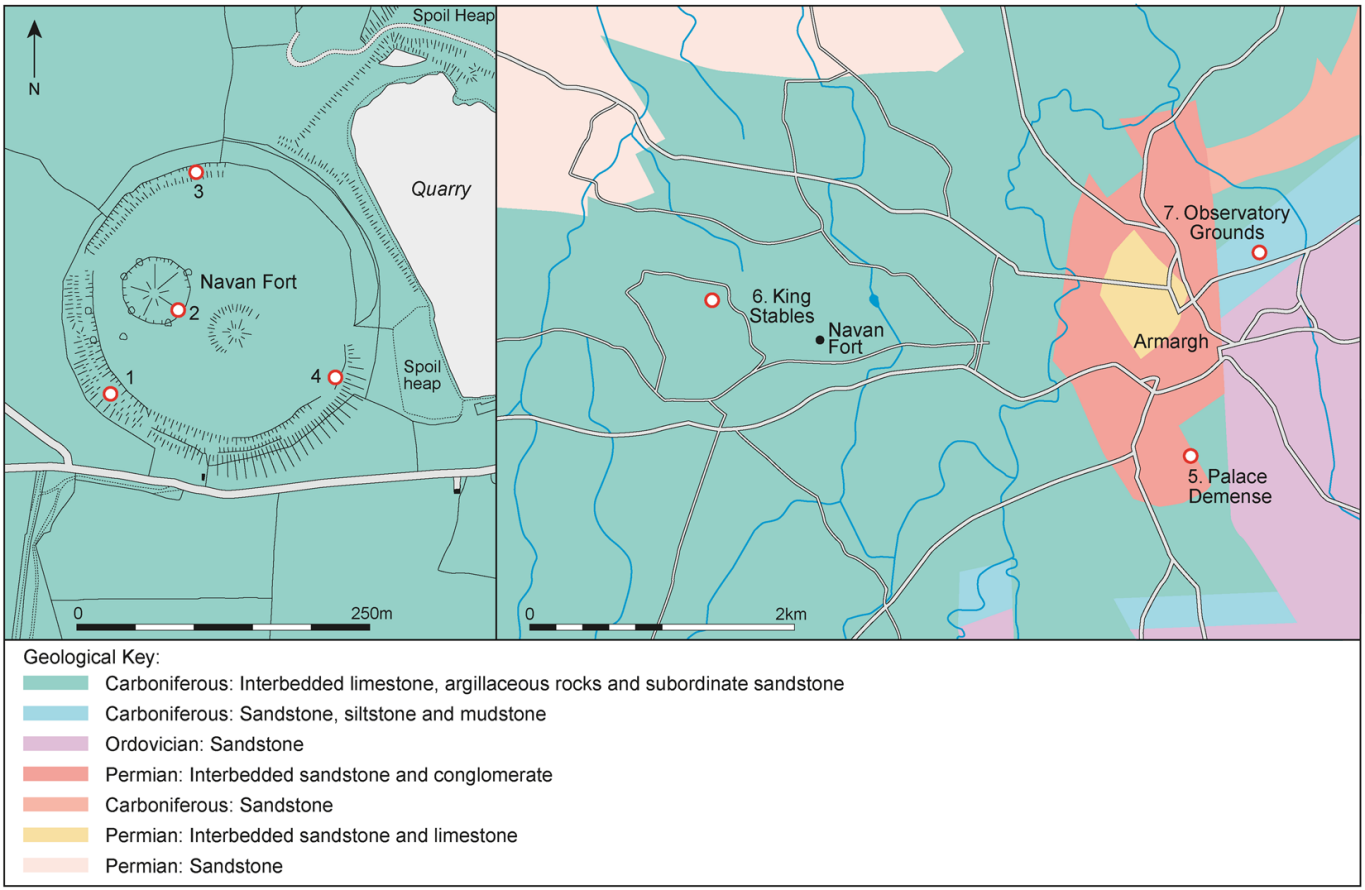
Map showing the location of the extracted plant samples (map created by Kirsty Harding following information from GSNI).

To establish whether non-local animals are present, it is imperative to determine the range of bioavailable strontium and sulphur in the vicinity of the site. Navan Fort is founded on Carboniferous limestone geology, covered by variable depths of glacial clays and sands^17^. Modern plant samples provide a useful means by which to determine the strontium biosphere range^18^. Snoeck *et al*.^19^ systematically analysed 88 plant samples from Northern Ireland to construct an initial strontium biosphere map. Only one sample location was relatively close to Navan Fort, on the Mid-Upper Ordovician Derryveeny formation, approximately 20 km southwest of the site. Analysis of shrubs and trees from this zone provided values between 0.7122 and 0.7128. Extrapolations from these data^19^ that relate to comparable lithologies surrounding the site suggest that values between 0.7099 and 0.7137 are likely at Navan Fort and its surrounding region (i.e. with an approximate radius of 20 km). As this local range involves extrapolation, seven plant samples were analysed from the immediate vicinity (within a 4 km radius) of the site (Fig. 5, Table 4). These results contrast with the expected bioavailable range defined by Snoeck *et al*.^19^. The results range from 0.7084 to 0.7096, which *is* consistent with expectation for the carboniferous limestone lithology on which the site is founded^20^ and this is thus defined as the local signature. However, there is no overlap with the previously published range^19^. As these samples from the immediate surroundings of the site are in close agreement they provide a coherent characterisation of the locality. This is not to say that the previously published map does not provide valid results, as it provides an important initial characterisation of Northern Ireland. Interpolations from comparable lithologies are necessary in mapping large areas such as this and these new data suggest that biosphere variation is likely to be more complex at a local scale, a feature that is becoming more commonly recognised in strontium isotope studies^21^. The published map may provide an indication of potential biosphere values in the broader region, but as only a single location was sampled in the vicinity of Navan Fort, further mapping is required.

**Table 4:** Sample details and results from the seven modern plants (lithological descriptions taken from the Geological Survey of Northern Ireland, see Fig. 2).

Sample	Description	Location	Lithology	^87^Sr/^86^Sr	δ^34^S
NAVP1	Blackthorn	Navan Fort (SW)	Carboniferous: Interbedded limestone, argillaceous rocks and subordinate sandstone	0.708642	7.9
NAVP2	Horse chestnut	Navan Fort (C)	Carboniferous: Interbedded limestone, argillaceous rocks and subordinate sandstone	0.708540	7.5
NAVP3	Thorny bush	Navan Fort (N)	Carboniferous: Interbedded limestone, argillaceous rocks and subordinate sandstone	0.709564	8.7
NAVP4	Unknown tree	Navan Fort (SE)	Carboniferous: Interbedded limestone, argillaceous rocks and subordinate sandstone	0.708612	7.9
NAVP5	Beech	Palace Demesne	Carboniferous: Interbedded limestone, argillaceous rocks and subordinate sandstone	0.709363	7.6 (leaf), 6.4 (stem)
NAVP6	Hawthorn	King’s Stables	Carboniferous: Interbedded limestone, argillaceous rocks and subordinate sandstone	0.708427	6.5
NAVP7	Thorny bush	Observatory grounds	Carboniferous: Sandstone, siltstone and mudstone	0.708669	0.2

Defining the local sulphur isotope signal is more challenging. The nature of variation is arguably better understood for Ireland than mainland Britain, as a result of the study by Zazzo *et al*.^15^. This study on sheep wool (keratin) recorded δ^34^S values from 5.8‰ to 17.0‰, with a significant negative correlation between sulphur values and distance from the west coast and lower values being commonplace in the midlands or east coast. Data were used to generate an indicative contour map for sulphur values in sheep wool for the majority of Ireland, with the lowest expected values being in the eastern midlands and the east coast (7–8‰) and the highest values being on the exposed west coast of County Mayo (15‰). The degree to which this map is valid for comparison with bone collagen values requires consideration. The diet-consumer tissue offsets for keratin and bone collagen are similar^22^. Modern studies have shown this offset to be slight, only around 0.5‰^13^. As the offset is slight and collagen and keratin are comparable in terms of their diet-consumer spacing, the map should be applicable to collagen analysis. However, as with the vast majority of biosphere maps, contours rely on interpolations based on a limited number of sample sites and biosphere variation is therefore likely to be more complex. The closest sampling location was Ballyhaise, c. 60 km inland from Navan Fort, which provided a mean value of 6.9‰ (+/−0.26‰).

The research by Zazzo *et al*.^15^ indicates that Navan Fort is in an area characterised by low δ^34^S values in the range of 7–8‰. However, given the contrasting results between the published strontium biosphere map and local plant strontium values, the same plant samples were also subject to sulphur isotope analysis. It is possible that modern plant values will be affected by industrial SO_2_ emissions^23^. However, reduced emissions in recent years and Ireland’s location on the western periphery of Europe with a predominantly southwesterly or westerly airflow should minimise any effect. Plant data are presented in Table 4. Sample values show very limited variation (6.4–8.7‰), excepting a single outlier (NAVP7 0.2‰). This aligns with the Zazzo map, suggesting low values are likely in animals raised in the vicinity of Navan Fort. The very low value (NAVP7) is markedly lower than any wool samples recorded by Zazzo *et al*.^15^. This suggests either that there are pockets in the local landscape that can produce even lower values, or that this sample from the observatory grounds has been subject to contamination by recent human activity and is not an accurate reflection of the prehistoric landscape baseline. Contamination is considered a possibility, as the observatory grounds are also quite close to a main road and Armagh town centre. Consequently, a relatively conservative local sulphur range of 6‰ to 9‰ can be suggested based on existing mapping data and new plant analyses, although the effects of modern contamination cannot be excluded.

### Identifying non-local fauna

Only eight (22%) of the 35 animals have values that fall within the local strontium range of 0.7084 to 0.7096. Assessing taxonomic differences in the representation of non-local animals is problematic in an unbalanced sample, but based on strontium isotope analysis alone, it is clear that neither of the caprines were locally raised. Only six of the pigs and two of the cattle have strontium values consistent with a local origin. However, lithological zones that would be expected to produce strontium values such as this are relatively common in Ireland and analyses of archaeological and modern plant samples from various locations have produced values in this range^19,24^. In addition, this strontium range also overlaps with the value for rainfall and seawater (0.70918), so values could result from sea-spray effects in coastal areas. Therefore, these animals could certainly have been raised elsewhere. Seven of these animals have sulphur isotope values ranging from 13.1 to 16.6‰ (NAV 37 was not subject to sulphur isotope analysis). These values are far higher than the expected local range of 6–9‰. The sulphur mapping suggests that none of the animals were raised in the locality of Navan Fort, as the entire dataset sits outside of the local sulphur range.

### Exploring origins

Strontium isotope values in the 35 animals from Navan Fort are exceptionally diverse. Although the strontium biosphere of the Republic of Ireland has only been partially mapped^25^ and mapping for Britain has not achieved optimal resolution (though it is improving rapidly^26^), it is clear that these animals have wide-ranging origins. Strontium values from 0.7071 to 0.7151 represent one of the largest ranges for any faunal dataset in the British Isles. Only Durrington Walls exhibits a wider range and this is in a far larger dataset of 138 animals^14,27^. Based on current published mapping this range encapsulated all strontium biosphere zones in the UK with the exception of very limited pockets of basalt lithology in the north of Ireland and highly radiogenic granitic lithology in northern Scotland^19,20^. While the wide-ranging values are certain to represent diverse origins, there are no clear clusters apparent within the data, whether using single or multiple isotope indices. This suggests that animals were not brought from distinct supply centres that supported the feasts, but rather that they were brought in small numbers from many locations.

According to the published strontium biosphere map^19^, all values in the dataset could be attained in Northern Ireland, but they are also consistent with origins throughout Ireland. Two pigs have highly radiogenic values (>0.714) that are well outside of the local range and that of the broader region^19^. Strontium values such as this are exceptionally rare in the northern Irish biosphere. Only four locations have produced plants in this range: two in the far south east of Ulster, one on the central northern coast and one near the western border of County Tyrone^19^. Of these, only the far south eastern location (County Down) could support the pig with the most radiogenic value (0.7151). None of these locations are close to Navan Fort (<50 km) and all represent relatively isolated pockets of radiogenic geology, uncharacteristic of a regional zone (they are therefore not presented as 0.714+ in the strontium biosphere map^17^). These animals must have been transported over distance to Navan Fort. They could have been raised in a restricted northerly area, principally feeding on resources from a constrained lithological zone. It is perhaps more likely that they derived from areas of older geology elsewhere, such as the metamorphic gneiss in limited areas of north western (Donegal), south eastern (Wexford) and central western (Galway) Ireland. Only one of the animals has a sulphur value (15.4‰). This is high and consistent with a coastal origin in western Ireland, making Donegal or Galway most likely.

The two lowest strontium values (0.7071) are the only two caprines that were analysed. A single pig also produced a similar result of 0.7072. These values are rare in the biosphere of Britain and Ireland. An origin in the tertiary basalts to the very north of Ireland (north and east of Lough Neagh) can be identified with confidence. The closest area to produce these values is south east of Lough Neagh (c. 30 km) but the Tertiary basalts dominate the north east of Northern Ireland and so origins cannot be further constrained using strontium alone. The caprines produced sulphur values of 15.6‰ and 16.5‰. These high values suggest a coastal origin in north Antrim (c. 100 km), as the closest basalt areas are in the zone that has produced the lowest sulphur values^15^. Sulphur isotope analysis of the pig failed to meet quality control criteria.

**Figure 6:**
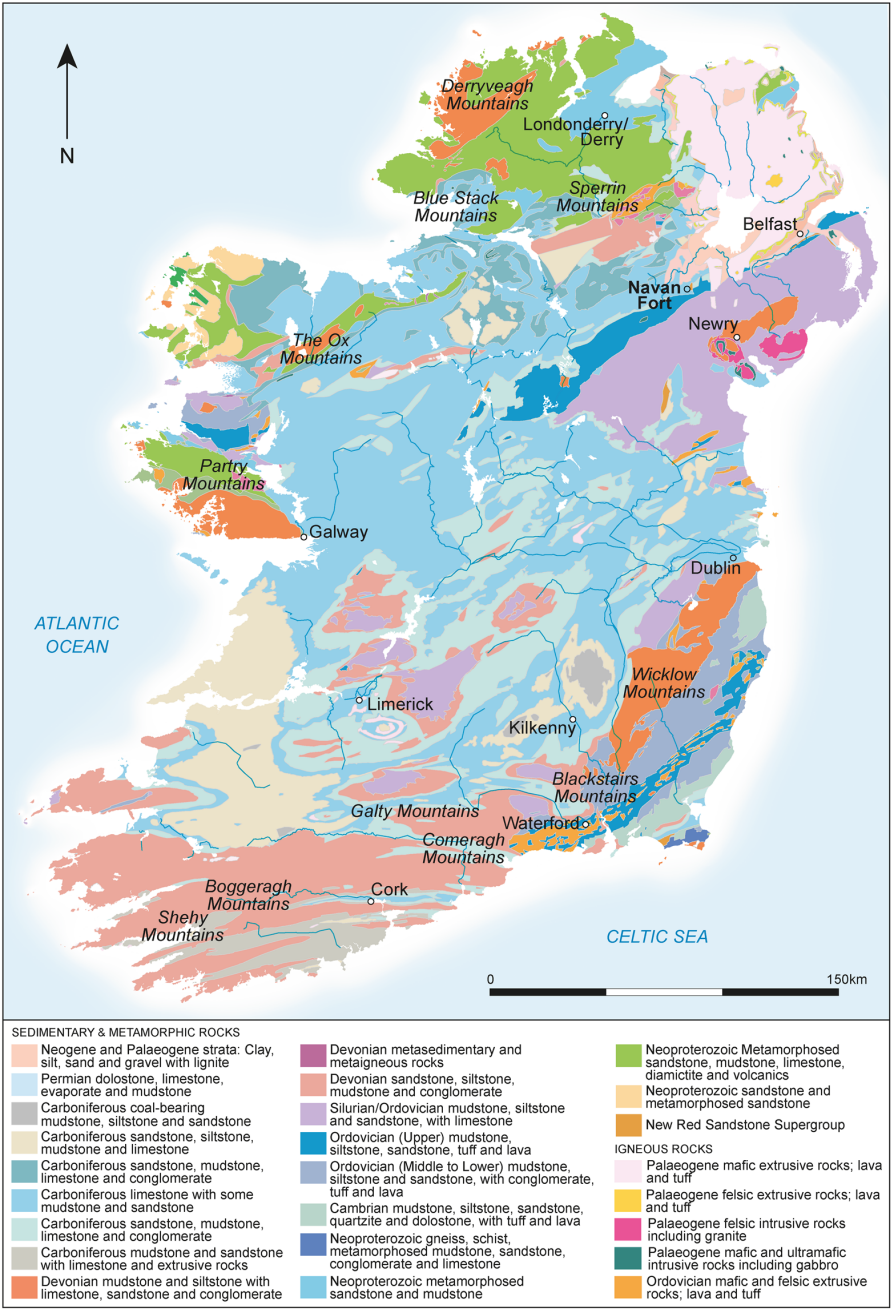
Geological map of Ireland showing the location of Navan Fort (map created by Kirsty Harding based on information from BGS/GSI/GSNI Bedrock Survey map).

Strontium biosphere mapping outside of Ulster has thus far principally focused on the central eastern area (County Meath), indicating that values between 0.708 and 0.713 are likely in this region^24,25^. A limited study has also focused on the Carrowkeel area of County Sligo in western Ireland, providing values of 0.7097 to 0.7119^28^. Ireland’s geology is dominated by carboniferous limestone (Fig. 6) that produces strontium biosphere values in the range of 0.7090 ± 0.0015 (2σ)^24,29^. Around half of the animals (12 pigs and 5 cattle) have values within this range and are very difficult to resolve geographically, as the limestone extends from the east to west coasts and from Lough Neagh in the north to County Waterford and Cork in the south. It is clear, however, that these animals came from several different regions of Ireland, as their sulphur values range from 13.1‰ to 16.9‰. Although this range is fairly restricted, it is not consistent with a single area of origin. In the wool dataset, the greatest range of mean sulphur values for individuals from the same location is 1.25‰^15^. Identifying likely origins is beyond the scope of the data at present, though the high sulphur values are suggestive of animals principally deriving from the west coast.

The remaining nine pigs and four cattle have strontium values between 0.7108 and 0.7123. These values are also very difficult to resolve geographically. They do not represent a homogenous ^87^Sr/^86^Sr biosphere zone and have sulphur isotope values ranging between 13.9‰ and 17.1‰, clearly indicating varied origins. Biosphere and archaeological research suggest that the strontium values are likely in parts of southern Ulster and in County Meath, predominantly on areas of Silurian and Ordovician mudstones^19,24,25^. These lithologies are present in various pockets across Ireland (Fig. 6). They are less common in the west and therefore the cattle sample with the highest sulphur value in the dataset may have originated from the zone of Ordovician and Silurian metasediments on the coastal area at the south of County Mayo.

**Figure 7:**
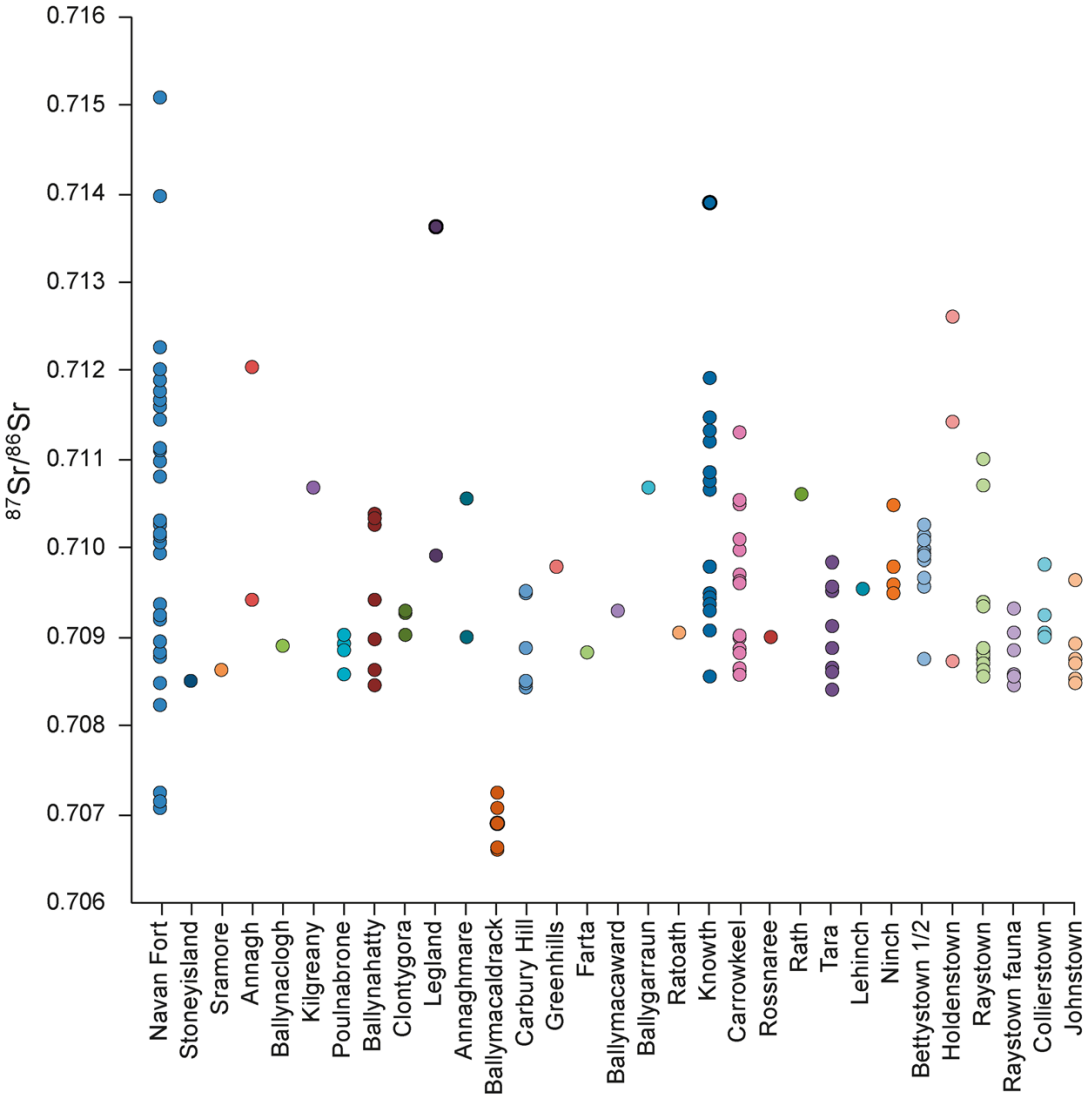
Strontium isotope values for humans and fauna from Ireland compared to the Navan Fort fauna (data from^19,24,28,29,51–58^).

This is the first isotope study focusing on the origins of archaeological domestic animals in Ireland and the first sulphur isotope study on any archaeological remains in Ireland that the authors are aware of. Consequently, comparative data is scarce and equifinality remains an interpretative problem. Identifying areas of origin with confidence remains beyond the scope of current data in most instances. Sulphur isotope analysis is becoming ever more useful in faunal provenancing when used in multi-isotope studies^14,30–32^, but detailed mapping is required for potential to be fulfilled and the interpretation of these data should be refined in the future. It is clear that the 35 animals were raised in wide-ranging locations across Ireland. The best comparisons can be made with research on human enamel and cremated bone from the Mesolithic to the early medieval period in Ireland (Fig. 7). This comparative dataset comprises a total of 132 human and faunal strontium values from 29 sites (samples from Tara sites have been amalgamated as a single site), which are plotted against the Navan Fort fauna in Fig. 7. The absolute range of values is far higher at Navan Fort than any other site. Navan Fort also comprises the two most radiogenic values in the entire dataset and has less radiogenic values than all sites with the exception of Ballymacaldrack (located on Antrim basalts). This provides clear evidence for the movement of large numbers of livestock from various area of Ireland to Navan Fort. The dataset also provides supporting evidence for origins in some instances. For example, the three low values (<0.7075) are comparable to those from Ballymacaldrack, supporting the assertion that these individuals may derive from the north Antrim basalts. There is no comparative archaeological data to assist interpretation of the sulphur data and therefore modern mapping must be used. Sulphur isotope results were less variable than strontium in relative terms and the consistently high values suggest that animals were frequently brought from the west of Ireland, but that central and eastern areas supplied few animals for the feasts. However, mapping resolution and the lack of comparative data means this interpretation can only be tentatively made at present.

The catchment of the feasts at Navan Fort must have been substantial. Animals provide a useful proxy for human movement, particularly in prehistory, when the vast majority of livestock would have been raised at a household level, rather than by specialist producers^33^. Domestic animals may even provide a better isotopic proxy for human origins than humans themselves. Humans are likely to acquire their food from a wider catchment than domestic animals and their teeth develop more slowly meaning analyses may represent a blended signal relating to different locations and phases in the individual’s life. This issue is even more acute in the analysis of cremated bone, which represents a longer-term averaged signal. Animals’ teeth develop rapidly and analysis therefore provides a more time-limited snapshot for origins, although foddering and transhumance can still complicate signals.

### Interpreting the carbon (δ^13^C) and nitrogen (δ^13^C) isotope data

The reconstruction of husbandry regimes through carbon and nitrogen isotope data requires the analysis of substantial numbers of animals and therefore only relatively fleeting comments can be made on this limited dataset.

The range of carbon and nitrogen values is not particularly wide but has marked outliers among the cattle and pigs. Wide-ranging values in pigs could result from variable foddering regimes in the same location. The spread of values in (herbivorous) cattle provides better evidence for different landscape origins, although local landscape variation and different foddering regimes cannot be discounted. Pigs generally have higher δ^15^N values than the cattle and sheep. This is a common pattern in carbon and nitrogen studies in temperate Europe^34–36^ and is consistent with pigs consuming some animal protein, potentially from meal scraps. However, the mean value for pigs is only 0.8‰ higher than that of cattle and therefore their diets are certain to be principally herbivorous. Even the two highest values of 7.6‰ are not consistent with a substantial input of animal protein. Higher values are commonplace in British pigs from the Neolithic^14^ to the medieval period, where nitrogen values above 12‰ have been attained^37^. The mean value of 6.3‰ is the same as for Iron Age pigs in the Pan-Irish study by Guiry *et al*.^38^, although pigs had a lower mean nitrogen value than cattle and caprines in this study. The low carbon values demonstrate that marine resources were not exploited for foddering the pigs. In spite of the wide-ranging origins of the pigs, it seems that feeding regimes show relatively limited variability and communities across Ireland were raising pigs on a largely herbivorous and entirely terrestrial diet, characteristic of a C3 ecosystem.

The low herbivore δ^15^N values (<7‰) indicate that none of the animals were raised in heavily manured terrain or on saltmarsh landscapes. By contrast the Guiry *et al*.^38^ study produced domestic herbivore values as high as 9.6‰ from Iron Age Ireland. The range of values is best explained by variable origins and underlying landscape variation in carbon and nitrogen isotope values. It is not possible to examine husbandry patterns relating to origins in such a limited dataset. There is no correlation between strontium and carbon (Pearson 0.31, sig. 0.875) or nitrogen (Pearson 0.115, sig. 0.553). Neither is there a correlation between sulphur and carbon (Pearson 0.142, sig. 0.471) or nitrogen (Pearson −0.52, sig. 0.793). However, it is noteworthy that the pig with the lowest strontium isotope value (0.7072), likely from the basalts of Antrim also had the lowest carbon (−22.9‰) and nitrogen (4.8‰) values in the dataset. This could relate to naturally low landscape values in this area (in which case low values would also be expected in the two caprines) or simply that this pig was raised on an entirely herbivorous diet in a landscape that had not been manured.

### Concluding comments

This research on Early Iron Age fauna from Navan Fort is the first isotope study to explore patterns of mobility through the analysis of animal remains in Ireland. The research demonstrates the interpretative potential of a multi-isotope approach in an Irish context and also the value of animals as a proxy for human movement. However, it also highlights the need for more comprehensive biosphere mapping. Current biosphere maps can be used as an indicative guide, but analysis of plants should be integrated into projects to provide more detailed local ranges.

The results provide clear evidence that communities in Iron Age Ireland were very mobile and that livestock were also moved over considerable distances. People brought animals from across Ulster and beyond to Navan Fort and it is likely that the great prehistoric regional centres of Ireland acted as lynchpins in the landscape and centres for large-scale connectivity. The bringing of animals from great distances to Navan can be explained in two different ways. Documentary evidence indicates that cattle raiding was as endemic feature of the medieval Ireland with some raids taking place on an inter-provincial scale^39^. Navan Fort is one of the principal settings of the Ulster Cycle of legendary tales which has at its core a tale of cattle raiding^40^. Such raiding, however, was primarily concerned with cattle while pig was primarily the food of feasting, as indicated in such legends as *The tale of Mac Da Thó’ pig*^41^. Feasting, almost invariably associated with sacrifice, was a social necessity of early societies where the slaughter of a large domesticate necessitated the consumption of a large amount of meat in a short period of time^11^. The results of the analysis of the pig bones from Navan provides evidence for such occasional feasting at the site, with participants bringing their pigs, for sacrifice and consumption, from a wide catchment area. Recent analysis of pig bones from Durrington Walls has provided similar evidence at a large Late Neolithic site^14^.

Equifinality and limited mapping remains a hurdle to precise interpretation of the isotope data and, therefore, identifying areas of origin with confidence is difficult. However, the diverse strontium values demonstrate wide-ranging origins and the vast majority of animals (potentially 31 of 35 analysed) were not raised in the vicinity of the site and the sulphur data points to many animals coming from western Ireland and fewer from eastern and central areas. The feasts at Navan Fort were clearly not supported by just one or two distinct supply centres, but rather utilised livestock from a much wider catchment. This is likely to represent animals (predominantly pigs) raised at a household level and brought to Navan Fort for the feast. Although current mapping precludes the identification of origins for most samples, some assertions can be made. Two sheep and a pig with very low strontium values are highly likely to have been raised in the basalt area of northern Ulster. The very high values are more difficult to pin to a location with confidence, but areas of County Donegal, Galway, Down, Tyrone and Antrim provide the most plausible places of origin. This provides new, direct evidence for both the scale and volume of mobility in later prehistoric Ireland. It is clear that Navan Fort had a vast catchment and that the influence of the site was far-reaching.

## Materials and Methods

A total of 30 faunal samples were analysed for ^87^Sr/^86^Sr, δ^34^S, δ^13^C and δ^15^N isotope analysis. In addition, five samples that were analysed for ^87^Sr/^86^Sr as part of a methodological study^42^ were used for comparison. Whilst this is a modest sample, it is the second largest published sample of fauna with combined strontium and sulphur data from the UK and Ireland (after^14^). Seven modern plant samples from the vicinity of the fort (Fig. 5) were analysed to provide an indication of the local ^87^Sr/^86^Sr and δ^34^S biosphere range. The plant samples were taken from deep rooting plants, such as leafy hedges or trees away from roads and farm buildings.

All faunal samples were from Site B at Navan Fort and came from layers associated with a series of superimposed round buildings found below the 40 m structure^17^. The buildings belong to the Late Bronze and Early Iron Age with most of the assemblage dating to between the 4^th^ and early 1^st^ century BC^17^. The samples comprised 24 pigs, 9 cattle and 2 caprines (Table 1). More stringent sampling constraints could be employed on the large pig assemblage. Second molars were analysed in all instances, with 27 being mandibular and two maxillary. A range of teeth had to be sampled for the cattle (dp4, M1, M2 and M3) and both caprine samples were M1. It was not possible to ensure that each sample represented a distinct individual by sampling elements from the same side in every instance. Efforts were made to sample enamel that developed at a similar period in the animal’s life for each taxon. The lower part of the cusp (i.e. closer to the root-enamel junction) was sampled for pigs. Depending on the length of time for mineralisation, this should provide a signal relating to approximately 6–10 months^43^. The mid cusp zone was sampled for the caprine M1s, providing a signal for origins in the first months of life. For cattle, lower cusp enamel was sampled for early developing teeth (dp4, M1) and mid-upper cusp enamel for second molars and upper cusp enamel for the M3, which develops last. Whilst the cattle samples will not all represent directly comparable periods in the animals’ lives, they are all broadly representative of early life origins and are therefore suited to addressing the objectives of the study. The lingual surface of the mesial lobe was targeted for pigs, but sometimes other surfaces were sampled due to poor preservation. The best preserved surfaces were sampled for other taxa.

Strontium (^87^Sr/^86^Sr) and sulphur (δ^34^S) isotope analysis were employed to explore the origins of the fauna. Carbon (δ^13^C) and nitrogen (δ^15^N) isotope analysis was undertaken to investigate husbandry regimes and to ensure that the other proxies could be interpreted in the context of origins rather than diet (e.g. to ensure that marine food sources did not impact on values). Initial sample preparation was undertaken at the Cardiff University BioArchaeology laboratory.

For strontium isotope analysis, enamel samples (10–50 mg) were cut from teeth and cleaned using a diamond saw and burr to remove all adhering dentine and at least 10 microns of the enamel surface. Samples were then transferred to a clean working area (class 100, laminar flow). Enamel samples were processed and analysed at the Applied Archaeological Sciences Laboratory and CREAIT MAF-IIC at Memorial University, Newfoundland. Plant samples were analysed at the National Environmental Isotope Facility.

Weighed enamel samples were cleaned with high purity (99%) acetone followed by deionized water (DI H_2_O, >18 MΩ) and dried. Enamel samples were dissolved in high purity HNO_3_ on a hot plate in clean 3 ml PFA vials (Saville, Eden Prairie, MN, USA) single distilled 8 M nitric acid (HNO_3_) on a hot plate. Strontium extraction from enamel samples used Sr.Spec and followed a common procedure for strontium purification where samples were loaded on to the resin in 1 ml 8 M distilled nitric acid. Matrix elements were then eluted in several washes (~3 ml) of 8 M HNO_3_ and purified strontium fractions were then collected for mass spectrometry using 2 ml DI water. Strontium isotope ratios were measured using a Thermo-Finnigan Neptune multi-collector inductively coupled mass spectrometer (MC-ICP-MS) in the CREAIT facility. All data was first corrected for on-peak blank intensities, then mass bias corrected using the exponential law and a normalization ratio of 8.375209 for ^88^Sr/^86^Sr^44^. Residual krypton (Kr) and rubidium (^87^Rb) interferences were monitored and corrected for using ^82^Kr and ^83^Kr (^83^Kr/^84^Kr = 0.20175 and ^83^Kr/^86^Kr = 0.66474; without normalization) and ^85^Rb (^85^Rb/^87^Rb = 2.5926), respectively. Analysis of NIST SRM 987 during the analytical session gave a ^87^Sr/^86^Sr value of 0.710292 ± 0.000007 (2σ n = 11) and all data is corrected a NIST SRM 987 values of 0.710248^45^. Total procedural blanks are typically <1% of typical sample ^88^Sr voltages and measurement of ^87^Sr/^86^Sr in NIST SRM 1400 (Bone Ash) gave a ^87^Sr/^86^Sr value of 0.713151, which is consistent with 0.713148, as published for this material^46^.

Plants were washed in DI water, dried and crushed to tea leaf consistency following Evans *et al*. (2010). Samples were then weighed into clean PFA (Perfluoroalkoxy) vials. Samples (0.1 g) were dissolved using a (MARS) microwave. Samples were pre-treated with Teflon distilled 8MHNO_3_ + ultra-pure H_2_O_2_, and placed on a hotplate overnight at 60 °C. The microwave container was then sealed and the samples microwaved at 170 °C for 20 minutes, before being transferred to Saville beakers. Additional H_2_O_2_ was added if the solution was darker than a straw yellow colour, and they were dried down. Samples were then converted to chloride form, using Teflon distilled 6 M HCl, dried down, and then taken up in 2.5MHCl ready for ion exchange separation. Strontium, from the plants samples, was separated using Dowex AG 50 × 8 cation resin. The strontium was loaded onto a single Re Filament with TaF^47^ and the isotope composition and concentrations were determined by Thermal Ionization Mass Spectrometry (TIMS) using a Thermo Triton multi-collector mass spectrometer. The samples were run using static mode and the International standard SRM 987 gave 0.710237 + − 0.000008 (2σ, n = 21). The data were corrected to a value of 0.710250.

For sulphur, carbon and nitrogen isotope analysis, sample preparation and collagen extraction was undertaken at the Cardiff University BioArchaeology laboratory. Compact bone from the mandibular body or ramus was sampled. Bone remodels and therefore provides an average signal for the years before death, in contrast to enamel which provides a more temporally constrained signal for early life. This means that the strontium and sulphur values do not relate to precisely the same time in the animals’ lives, but in short-lived domesticates the temporal range is certain to overlap. The collagen-extraction protocol followed a modified version of the Longin method^48^. Approximately 0.5 g of bone was sampled from each specimen using a precision drill with a diamond rotary wheel attachment. The cortex was abraded using a burr to remove adhering contaminants. Each sample was then demineralised in 8 ml of 0.5 M HCl at 4 °C. Demineralised specimens were thoroughly rinsed in DI water and then gelatinized in a pH3 solution of HCl at 70 °C for 48 hours. The supernate containing the soluble collagen was collected using an 8μm ezee-filter (Elkay, Basingstoke) and transferred to a polypropylene test tube for freeze-drying. Plant leaf samples were cryogenically milled before mass spectrometry.

For δ^13^C/δ^15^N isotope analysis, 0.75 mg of collagen was weighed in duplicate for each sample. For δ^34^S isotope analysis, 8 mg of collagen was weighed in duplicate, with the addition of V_2_O_5_ to aid combustion_._ Plant samples were weighed to 2 mg in triplicate for δ^34^S isotope analysis. Isotope ratios of carbon, nitrogen and sulphur were measured by continuous flow-elemental analyser-isotope ratio mass spectrometry (CF-EA-IRMS). Sulphur isotope analysis was undertaken at the National Environmental Isotope Facility  at the British Geological Survey. For collagen samples, the instrumentation comprises an elemental analyser (Flash/EA) coupled to a ThermoFinnigan Delta Plus XL isotope ratio mass spectrometer via a ConFlo III interface. Plant samples were analysed using a ThermoFinnigan EA IsoLink coupled to a Delta V Plus isotope ratio mass spectrometer via a ConFlo IV interface. Carbon and nitrogen isotope analysis was undertaken at the School of Earth and Ocean Sciences, Cardiff University using a Flash 1105 elemental analyser coupled to a ThermoFinigan Delta V Advantage. Carbon, nitrogen and sulphur isotope ratios (δ^13^C, δ^15^N and δ^34^S) are reported in per mil (‰) relative to VPDB, AIR and VCDT standards respectively. Analysis of all samples complied with collagen quality control criteria for sulphur^49^ (C:S ratio of 600 ± 300; N:S ratio of 200 ± 100, Table 2), with the exception of two samples (NAV24 and NAV30) for which the sulphur value was excluded. Samples had an atomic C:N ratio between 2.9 and 3.6 and were therefore considered to be sufficiently well preserved to yield reliable δ^13^C and δ^15^N values^50^. The only exception was NAV24 and these isotope values were therefore omitted. Sulphur isotope ratios were calibrated against an in-house powdered gelatine standard (M1360P from British Drug Houses), providing a mean 1σ reproducibility of 0.24 and using a secondary check in-house collagen material. M1360P was also used for the calculation of %S and was calibrated to VCDT using IAEA standards S1 and S2. Plant δ^34^S samples were corrected using S1 = −0.03 vs vCDT & S2 = 22.7 vs VCDT, and SOIL A = 0.126%S. Duplicate sample reproducibility was variable (mean 1σ reproducibility of 0.49 for collagen and 0.28 for plants) but no samples were excluded on this basis due to only large differences in sulphur values being interpreted. Carbon and nitrogen isotope ratios were calibrated against caffeine (laboratory grade, 98.5%, Acros Organics, lot A0342883) and an in-house supermarket gelatine standard, which is calibrated against IAEA-600 (^13^C and ^15^N), IAEA-CH-6 (^13^C), and IAEA-N-2 (^15^N). The 1σ reproducibility was 0.08 for δ^13^C and 0.07 for δ^15^N. Caffeine was used to calculate %N and %C (28.85% N and 49.48% C, calculated from the molecular formula C_8_H_10_N_4_O_2_).

## Data Availability

All data generated or analysed during this study are included in this published article.
